# Diverse Alterations of Glomerular Capillary Networks in Focal Segmental Glomerular Sclerosis

**DOI:** 10.1016/j.ekir.2022.03.007

**Published:** 2022-03-14

**Authors:** Megumi Morita, Akiko Mii, Fumihiko Yasuda, Yusuke Arakawa, Tetsuya Kashiwagi, Akira Shimizu

**Affiliations:** 1 Department of Nephrology, Adachi Iriya Toneri Clinic, Tokyo, Japan; 2 Department of Nephrology, Nippon Medical School, Tokyo, Japan; 3 Department of Nephrology, Meirikai Chuo General Hospital, Tokyo, Japan; 4 Department of Analytic Human Pathology, Nippon Medical School, Tokyo, Japan

**Keywords:** biopsy, capillaries, computer-assisted image analysis, endothelial cells, focal segmental glomerulosclerosis, glomerular filtration rate

## Abstract

**Introduction:**

Focal segmental glomerular sclerosis (FSGS) is caused by podocyte injury. It is characterized by obliteration of glomerular capillary tufts with increased extracellular matrix (ECM). Altered communication between podocytes and glomerular endothelial cells (ECs) contributes to sclerosis progression. We focused on EC injury in the FSGS.

**Methods:**

A total of 29 FSGS and 18 control biopsy specimens were assessed for clinicopathologic characteristics. CD34 (a marker for EC)-positive capillaries and ECM accumulation were evaluated quantitatively for each variant using computer-assisted image analysis.

**Results:**

The estimated glomerular filtration rate (eGFR) in the FSGS group was significantly lower than that in the control group. The frequency of FSGS variants was 51.7% for cellular; 13.8% for perihilar (PH), tip, and not otherwise specified (NOS); and 6.9% for collapsing. Regarding sclerotic lesions in all FSGS, narrowing or loss of CD34-positive capillaries was observed. Electron microscopy results showed loss of fenestrae, subendothelial space enlargement, and cytoplasmic swelling, indicating EC injury. Computer-assisted image analysis revealed significantly smaller areas of glomerular capillaries in FSGS with or without sclerotic lesions, with increased ECM. Moreover, in comparison with each variant, narrowed capillaries and ECM accumulation were most prominent in the collapsing variant, whereas the tip variant had the least change.

**Conclusion:**

EC injury was observed in all FSGS cases, not only in sclerotic lesions but also in nonsclerotic lesions. Severity of EC injury may vary in each variant due to diverse alterations of glomerular capillary networks.

## Graphical abstract

**Figure:**
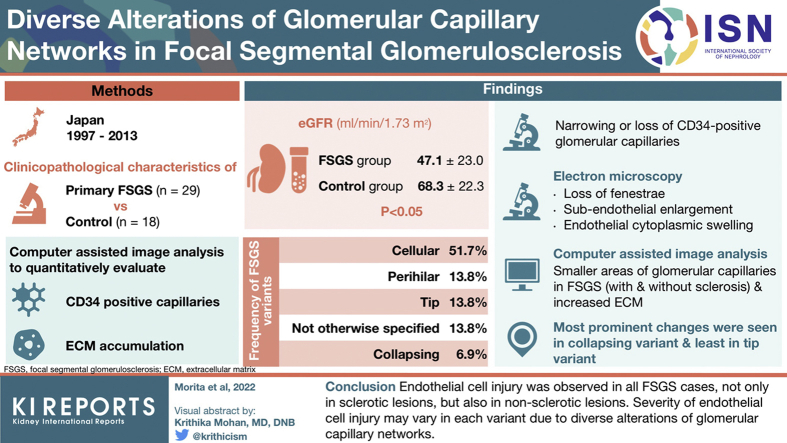

FSGS is a common primary glomerular disorder representing a risk factor for end-stage renal failure.^1^ Since D’Agati *et al.*^2^ proposed the Columbia Classification of FSGS in 2004, many studies have shown that clinical features and prognosis of FSGS vary greatly depending on the FSGS variant.^3^, ^4^, ^5^, ^6^, ^7^, ^8^, ^9^, ^10^, ^11^ These studies suggested diverse pathogeneses of the disease. Because FSGS is called “podocyte disease” or “podocytopathy” along with minimal-change nephrotic syndrome (NS), the initial target is podocyte injury.^1^^,^^12^^,^^13^ Various constituent cells of the kidney have been reported to be involved in progression of the process. These include podocyte injury to segmental sclerosis in FSGS, phenotypic changes in parietal epithelial cells, and crosstalk between glomerular epithelial and tubular epithelial cells and between glomerular endothelial or mesangial cells and podocytes.^12^, ^13^, ^14^

Podocytes regulate proliferation and function of glomerular ECs through vascular endothelial growth factor A and endothelin-1 to maintain glomerular capillary loop homeostasis. There have been reports that conditional knockout of vascular endothelial growth factor A in podocytes caused EC death and thrombotic microangiopathy.^15^ Conversely, overexpression of vascular endothelial growth factor A in podocytes caused collapsing glomerulopathy, as seen in HIV nephropathy.^16^ Daehn *et al.*^14^ reported that endothelin-1 released from damaged podocytes promoted mitochondrial oxidative stress and dysfunction of adjacent ECs. Eventually, this EC injury further promoted podocyte apoptosis. They also indicated that targeting this interaction between podocytes and ECs may be a potential therapeutic target for FSGS.^14^ A randomized, double-blind, active-controlled study (DUET study) compared the effects of sparsentan, a dual antagonist of endothelin A and angiotensin II type 1 receptors, and irbesartan, an angiotensin II type 1 receptor blocker. That study confirmed that sparsentan, administered for 8 weeks in patients with primary FSGS, significantly reduced proteinuria compared with irbesartan.^17^

However, detailed studies of EC injury in FSGS cases using human renal biopsy specimens are still limited. Van de Lest *et al.*^18^ showed increase in glomeruli with endothelin receptor A-positive ECs in 39 FSGS biopsy specimens. This was associated with nephrin loss and increased 8-oxoguanine–positive staining, a DNA lesion caused by oxidative damage, and increased proteinuria levels.^18^ Taneda *et al.*^19^ used electron micrographs of FSGS renal biopsy specimens to measure subendothelial widening as a marker for EC injury. They reported that this widening was associated with poor remission rates and decreased eGFR.^19^ Murer *et al.*^20^ reported increased endothelin expression in the glomeruli in steroid-resistant FSGS specimens from children. The findings of biopsy specimens are difficult to prove because of the general features of FSGS and the assessments are often substituted by semiquantitative ones, which do not exclude investigator bias. We have previously quantitatively evaluated EC injury to membranous nephropathy with segmental sclerosis using computer-assisted morphometric analysis. We also reported that EC injury was observed in all cases of membranous nephropathy, and EC injury was more severe in cases with segmental sclerosis than in cases without segmental sclerosis.^21^ In this current study, we quantitatively evaluated and assessed EC injury in FSGS using a computer-assisted morphometric analysis method in human renal biopsy specimens.

## Methods

### Ethics

This study was performed in accordance with the Declaration of Helsinki and was approved by the Ethics Review Committee of Nippon Medical School (approval number: B-2020-167). Informed consent to use the specimens and clinical data for research was obtained from all patients or from their parents or legal guardians if patients were under 18 years of age.

### Case Selection

A total of 29 cases diagnosed with having primary FSGS were retrospectively identified from a series of renal biopsies performed at the Nippon Medical School between 1997 and 2013. All cases had no evidence of secondary causes of FSGS, such as reflux nephropathy; surgical ablation; solitary kidney; sickle cell anemia; viral infections such as HIV and parvovirus B19; and family history of renal disease. In addition, 18 patients diagnosed with having minor glomerular abnormalities (10 with NS and 8 with persistent proteinuria) were included in the control group for computer-assisted morphometric analysis, with no significant difference in age. We examined the clinicopathologic characteristics in these patients.

### Clinical and Pathologic Data

Data of age, sex, time to biopsy, NS, and eGFR in all patients at the time of biopsy were retrospectively collected from their clinical records. The time to biopsy was defined as the time from the first abnormal urinary symptom noted in the clinical record to biopsy.

Renal biopsy specimens were evaluated by light microscopy, immunohistochemistry, and electron microscopy. Formalin-fixed, paraffin-embedded tissue sections for light microscopy were prepared and stained with periodic acid–Schiff and periodic acid–methenamine silver. Immunostaining for CD34 (NU-4A1, Nichirei Bioscience, Tokyo, Japan) was performed to detect ECs and evaluate morphologic alterations of the glomerular capillaries. In detail, FSGS cases were evaluated. Moreover, the FSGS lesions were characterized using the Columbia criteria for the classification of FSGS.^2^

Electron microscopy was performed in all cases of FSGS. Ultrathin sections from Epon-embedded tissue samples were fixed in 2.5% glutaraldehyde, postfixed in 1% osmium tetroxide, stained with uranyl acetate and lead citrate, and examined with a Hitachi H7500 transmission electron microscope (Hitachi, Ibaraki, Japan).

### Computer-Assisted Morphometric Analysis

Using a computer-assisted image analyzer (Win Roof, Mitani Corp., Japan), we assessed the whole glomerular tuft area, number and area of the glomerular capillaries, and glomerular ECM area of each glomerulus in immunostained sections for CD34 (glomerular capillaries) and periodic acid–Schiff stain (ECM). Area of the glomerular tuft was extracted by enclosed area of the dotted line. Area of the glomerular capillaries was measured as the entire space enclosed by CD34-positive ECs in the glomeruli and automatically calculated each circle number as the number of the glomerular capillaries. Area of glomerular ECM was measured as periodic acid–Schiff stain–positive area in the glomeruli. The average area of the glomerular capillaries was calculated by dividing the entire area of the glomerular capillaries by the number of the glomerular capillaries.

### Statistical Analysis

All statistical analyses were performed using EZR (Saitama Medical Center, Jichi Medical University, Saitama, Japan), which is a graphical user interface for R (The R Foundation for Statistical Computing, Vienna, Austria). More precisely, it is a modified version of R commander frequently used in biostatistics designed to add statistical functions.^22^

Data are expressed as mean ± SD. Statistical analysis was performed using nonparametric test methods, such as Fisher exact test (for categorical variables), Mann-Whitney *U* test (for continuous variables between 2 groups), and Kruskal-Wallis test (for continuous variables between 3 groups or 6 groups), where appropriate. For *post hoc* analysis, the Steel–Dwass test was used to compare the variables of each group. Spearman's rank correlation coefficient was used to evaluate the correlation between 2 continuous variables. In all tests, statistical significance was set at *P* < 0.05.

## Results

### Clinical Characteristics

Table 1 shows the clinical features of the 29 patients with primary FSGS and 18 controls. The average age at biopsy was 50.0 ± 18.0 years, ranging from 16 to 83 years. All patients were Japanese, and the male-to-female ratio was 65.5:34.5. Proteinuria (≥0.5 g/g creatinine) was observed in all patients, and 75.9% of these patients had NS (massive proteinuria ≥ 3.5 g/d) and hypoalbuminemia (albumin ≤ 3.0 mg/dl). The eGFR in the FSGS group (47.1 ± 23.0 ml/min per 1.73 m^2^) was significantly lower compared with that in the control group (68.3 ± 22.3 ml/min per 1.73 m^2^, *P* < 0.05). There were no significant differences in age, sex, time to biopsy, or frequency of NS between the 2 groups.

**Table 1:** Clinical features at the time of biopsy

Characteristics	Control (*n =* 18)	FSGS (*n* = 29)	*P* value
Age (yr)	59.5 ± 10.3 (35-76)	50.0 ± 18.0 (16-83)	ns
Male/female (*n*)	10 / 8	19 / 10	ns
Time to biopsy (mo)	14.5 ± 27.0	57.7 ± 82.0	ns
Nephrotic syndrome (%)	55.6	75.9	ns
eGFR (ml/min per 1.73 m^2^)	68.3 ± 22.3	47.1 ± 23.0	<0.01

eGFR, estimated glomerular filtration rate; FSGS, focal segmental glomerular sclerosis; ns, not significant.

Quantitative variables are mean ± SD.

According to the Columbia classification criteria for FSGS lesions,^2^ the frequency of the 5 variants among the 29 FSGS cases was 51.7% (*n =* 15) for cellular; 13.8% (*n =* 4) for PH, tip, and NOS; and 6.9% (*n =* 2) for collapsing (Table 2). Owing to the small number of the cases, statistical analysis could not be applied to the characteristics of each variant. Time to biopsy was defined as the time from the first abnormal urinary symptom to biopsy, and the tip variant had a shorter time than did the other variants. The collapsing variant had only 2 adult cases. However, both cases occurred in childhood. The time to biopsies was long because data from the second biopsy were used in this study. In the PH variant, 2 cases were also on their second or third biopsy. NS was diagnosed in >90% of the patients with cellular, tip, and collapsing variants, but only in 1 of 4 patients with PH variants. Regarding renal function, both cases with collapsing variants tended to have a lower eGFR value compared with those with the other variants.

**Table 2:** Clinical features of each FSGS variant at the time of biopsy

Characteristics	Tip (*n =* 4)	Collapsing (*n =* 2)	Cellular (*n =* 15)	Perihilar (*n =* 4)	NOS (*n =* 4)
Age (yr)	54.0 ± 13.0 (37–74)	23.0 ± 1.0 (22–24)	61.0 ± 13.0 (32–83)	32.0 ± 15.0 (18–56)	46.0 ± 20.0 (16–70)
Male/female (*n*)	3/1	0/2	9/6	3/1	4/0
Time to biopsy (mo)	2.0 ± 2.3	156.0 ± 0.0	20.0 ± 47.6	152.0 ± 99.12	135.0 ± 72.7
Nephrotic syndrome (%)	100	100	93.3	25	50
eGFR (ml/min per 1.73 m^2^)	72.5 ± 6.6	23.1 ± 4.7	44.2 ± 24.1	50.6 ± 9.5	41.0 ± 14.7

eGFR, estimated glomerular filtration rate; FSGS, focal segmental glomerular sclerosis; NOS, not otherwise specified.

Quantitative variables are mean ± SD.

### The Alternations of Glomerular Capillary Network in Each Variant of FSGS

**Figure 1:**
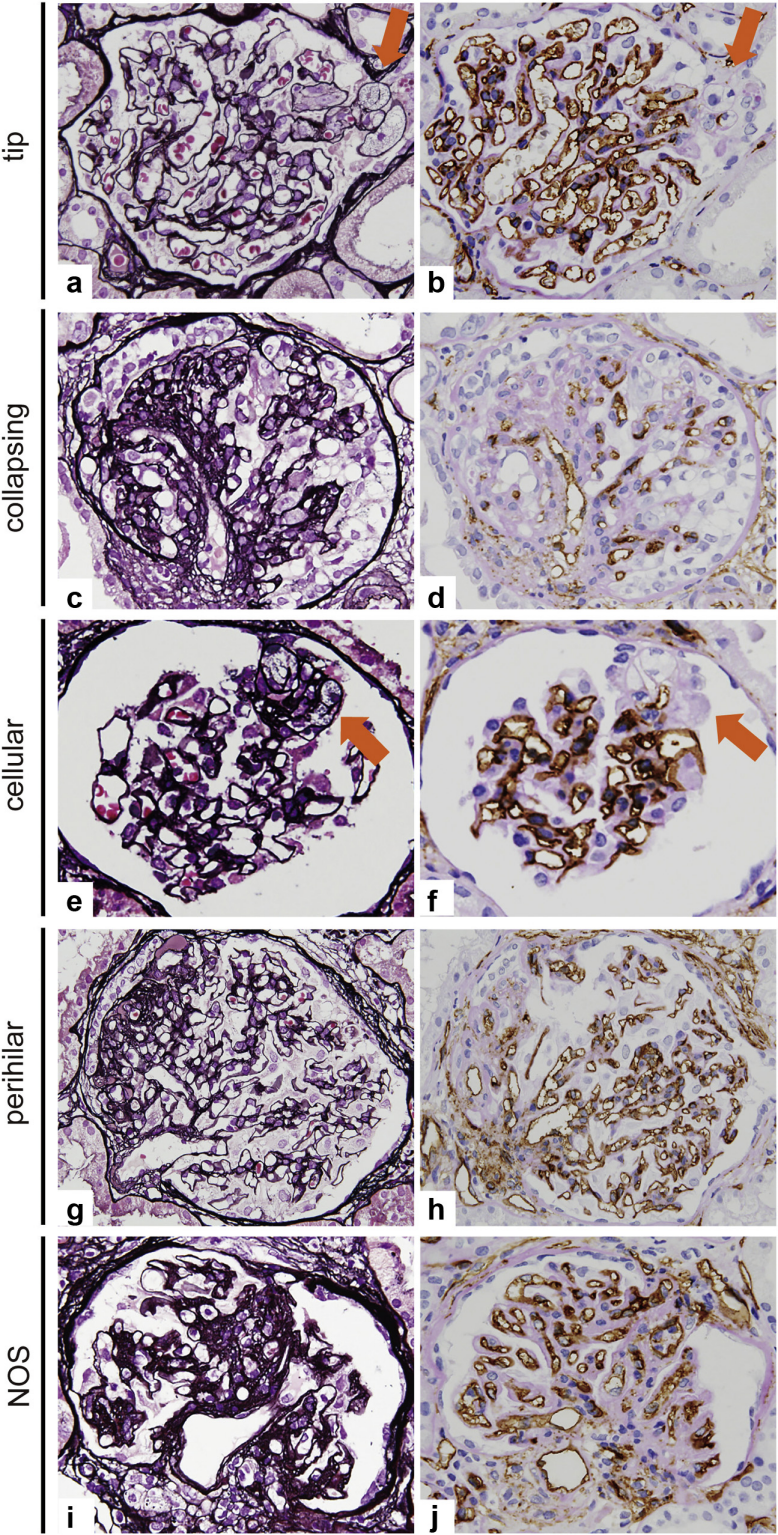
Glomerular capillary alterations in each variant of FSGS. In the case of the tip variant (a, b), the tip lesions show disappearance of CD34+ glomerular endothelial cells and loss of glomerular capillary lumens with foam cell infiltration (arrow). In the collapsing variant (c, d), the glomerulus shows global loss of CD34+ glomerular endothelial cells with hyperplastic podocytes and collapsed glomerular capillaries. In the cellular variant (e, f), loss of glomerular capillary lumens with disappearance of CD34+ endothelial cells is observed in the segmental lesion with endocapillary proliferation (arrow). In the cases of PH variant (g, h) and NOS variant (i, j), CD34+ endothelial cells disappeare in the segmental sclerosis lesion and ECM accumulated around the collapsed capillaries (a, c, e, g, i: PAM stain, ×600; b, d, f, h, j: CD34 stain, ×600). ECM, extracellular matrix; FSGS, focal segmental glomerular sclerosis; NOS, not otherwise specified; PAM, periodic acid–methenamine silver; PH, perihilar.

Glomerular capillary network was clearly found by immunostaining for CD34 (a marker for ECs) with periodic acid–Schiff counterstain compared with periodic acid–methenamine silver staining. Representative images in each variant of FSGS are shown in Figure 1a to j. In the tip variant, CD34+ glomerular capillary lumina were lost in the tip lesion (Figure 1a). However, CD34+ glomerular ECs and capillary lumen were preserved in other glomerular capillaries outside the tip lesion (Figure 1b). In contrast, in the collapsing variant, extensive loss of CD34+ glomerular ECs with podocyte hypertrophy and collapsed glomerular capillaries was observed, which indicated severe glomerular endothelial injury globally (Figure 1c and d). In the other variants, CD34+ ECs were also lost in the segmental sclerosis lesion, and ECM accumulated around the collapsed capillaries (Figure 1e–j). A common finding among the cellular, NOS, and PH variants was that narrowing of the glomerular capillaries was found not only in the segmental sclerotic area but also in the areas outside of the segmental glomerular lesions.

### Ultrastructure of Glomerular Capillaries in FSGS

**Figure 2:**
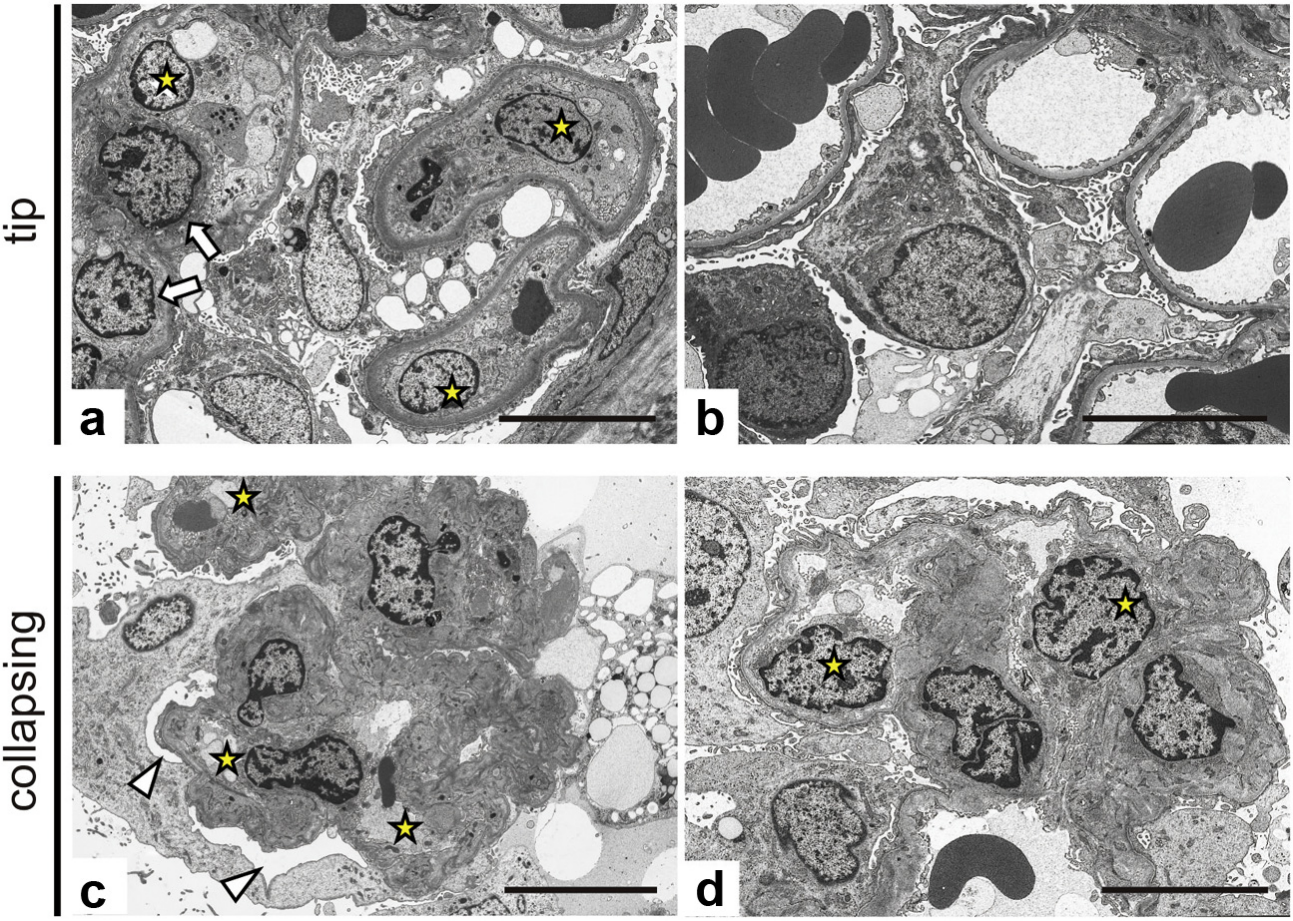
Ultrastructure of endothelial cell injury in the tip and collapsing variant. In the tip lesion of the tip variant (a), swelling of the glomerular endothelial cells (arrow), loss of fenestrae, and effacement of foot processes of podocytes are observed, along with infiltration of macrophages (star). In lesions other than the tip lesion (b), the endothelial damage is relatively mild with maintained fenestrae, mild swelling of the endothelial cells, and hardly detectable widening of the subendothelial space. The collapsing variant (c, d) shows podocyte hypertrophy and vacuolization with expansion of the subpodocyte space (arrowhead) and effacement of foot processes. Narrowed glomerular capillaries (star) are seen with wrinkling of the GBM, indicating collapse of the glomerular capillaries. In the remaining capillary lumens, endothelial cells become swollen (star in d) with irregular loss of fenestra and mild widening of subendothelial spaces. Bar = 10 μm. GBM, glomerular basement membrane.

Representative images of electron microscopy (EM) in the tip and collapsing variants are shown in Figure 2a to d. In the tip lesion of the tip variant, swelling of glomerular ECs and loss of fenestrae in addition to podocyte foot process effacement were observed along with macrophage infiltration (Figure 2a). In contrast, in lesions other than the tip lesions, although podocyte foot process effacement was detected, fenestration of ECs was maintained, swelling of ECs was mild, and subendothelial space was hardly enlarged. These findings suggested that EC injury other than the tip lesions was mild (Figure 2b).

In contrast, in the collapsing variant of FSGS, we observed hypertrophy and vacuolation of podocytes with subpodocyte space expansion and loss of foot processes. Furthermore, extremely narrowed and collapsed glomerular capillaries were seen with wrinkling of the glomerular basement membrane (Figure 2c and d).

Ultrastructurally, even in varying degrees of variants of FSGS, glomerular EC injuries along with podocyte damages might have been developed in FSGS. Findings of EM also suggested that severity of glomerular endothelial damage differed among FSGS variants without performing quantitative analysis. Therefore, we further performed quantitative evaluation.

### Visualization and Quantitative Analysis of Glomerular Capillary Network

**Figure 3:**
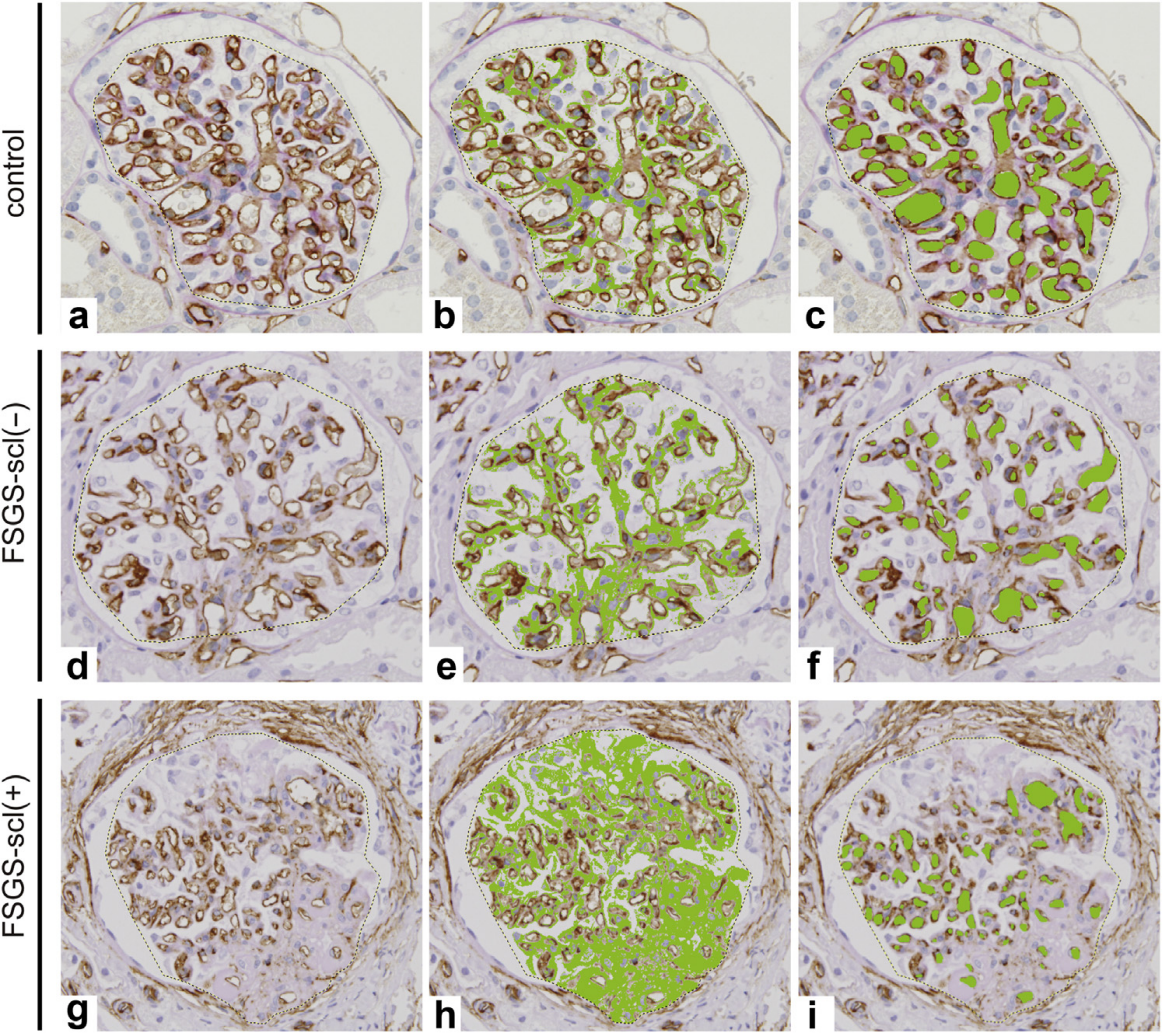
Visualization and quantitative analysis of glomerular capillary network using computer-assessed image analyzer. In the control glomeruli (a–c), FSGS-scl (−) glomeruli (d–f), and FSGS-scl (+) glomeruli (g–i), area of the glomerular tuft (dotted lines a, d, g), ECM area (b, e, h green areas), and area and number of the glomerular capillaries (c, f, I, green areas) are evaluated using a computer-assessed image analyzer. The glomerular capillary network lined with CD34+ glomerular endothelial cells (a) and capillary area (c) is well preserved without ECM accumulation (b). In contrast, narrowing and decrease of glomerular capillaries with ECM accumulation are apparent in FSGS-scl (+) glomeruli (g–i). Even in the FSGS-scl (−) glomeruli (d–f), glomerular capillary lumens are clearly narrowed compared with the control glomeruli. ECM, extracellular matrix; FSGS, focal segmental glomerular sclerosis; FSGS-scl(−), glomeruli without sclerotic lesions in FSGS; FSGS-scl(+), glomeruli with sclerotic lesions in FSGS.

To visualize the glomerular capillary area and ECM accumulation, we used the computed-assisted morphometric analysis. The glomeruli with cross-sections of the whole glomerular tuft containing vascular poles were analyzed. The samples consisted of 96 glomeruli in control cases (Figure 3a–c), 131 glomeruli without sclerotic lesions in FSGS cases (FSGS-scl[−]) (Figure 3d–f), and 23 glomeruli with sclerotic lesions in FSGS cases (FSGS-scl[+]) (Figure 3g–i). In the control cases, CD34+glomerular capillaries were well dilated, and there was no ECM accumulation (Figure 3b and c). In contrast, narrowing and reduction of the glomerular capillaries with accumulation of ECM was more pronounced in FSGS-scl(+) glomeruli than in the other groups (Figure 3h and i).

**Figure 4:**
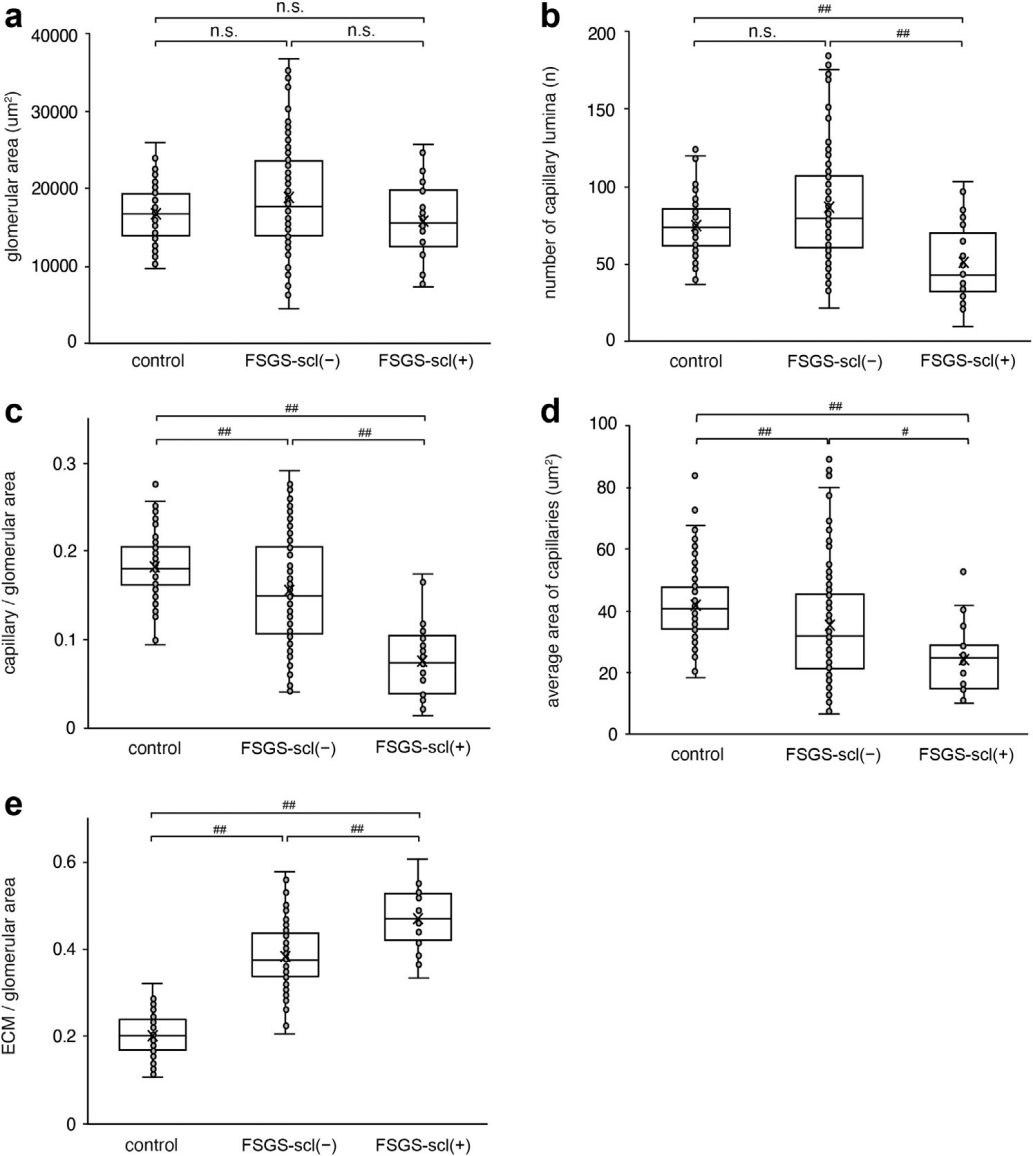
The quantitative analysis of the glomerular capillary network in FSGS with or without sclerosis lesion. The glomerular tuft area (a), the number of glomerular capillaries (b), the glomerular capillary/glomerulus area ratio (c), the average area of capillaries (d), and the glomerular ECM/glomerulus area ratio (e) are compared among FSGS-scl(+) glomeruli, FSGS-scl(−) glomeruli, and control glomeruli. #*P* < 0.05, ##*P* < 0.01 Kruskal-Wallis test and for each group using Steel–Dwass test. The box corresponds to the first quartile, median (horizontal bar in the box), and third quartile, and the whiskers extend from minimum to maximum values. ECM, extracellular matrix; FSGS, focal segmental glomerular sclerosis; FSGS-scl(−), glomeruli without sclerotic lesions in FSGS; FSGS-scl(+), glomeruli with sclerotic lesions in FSGS; n.s., no significance.

Then, we quantified the area of the glomerular tuft and capillaries, and number of capillaries, and compared them among the 3 groups (Figure 4a–e). The glomerular tuft area was not significantly different among the 3 groups. However, there were some variations in the FSGS-scl(−) group (Figure 4a). It is suggested that glomerular hypertrophy may have occurred due to hyperfiltration in some glomeruli without sclerosis. In the FSGS-scl(−) group, although the area of the glomerular capillaries was significantly smaller and the ECM was increased compared with that in the control group, there was no significant difference in the number of capillary lumina (Figure 4b–e). In the FSGS-scl(+) group, the area and number of glomerular capillaries were significantly smaller and ECM accumulation was more prominent than in the other groups (Figure 4b–e). In addition, the average area of capillaries was also significantly smaller than in the other groups even though both the area and number of capillaries were decreased. These results suggested that alteration of glomerular capillary network was most pronounced in the FSGS-scl(+) group and that narrowing of the capillary lumina has begun, even in the glomeruli without sclerotic lesions in the FSGS cases.

**Figure 5:**
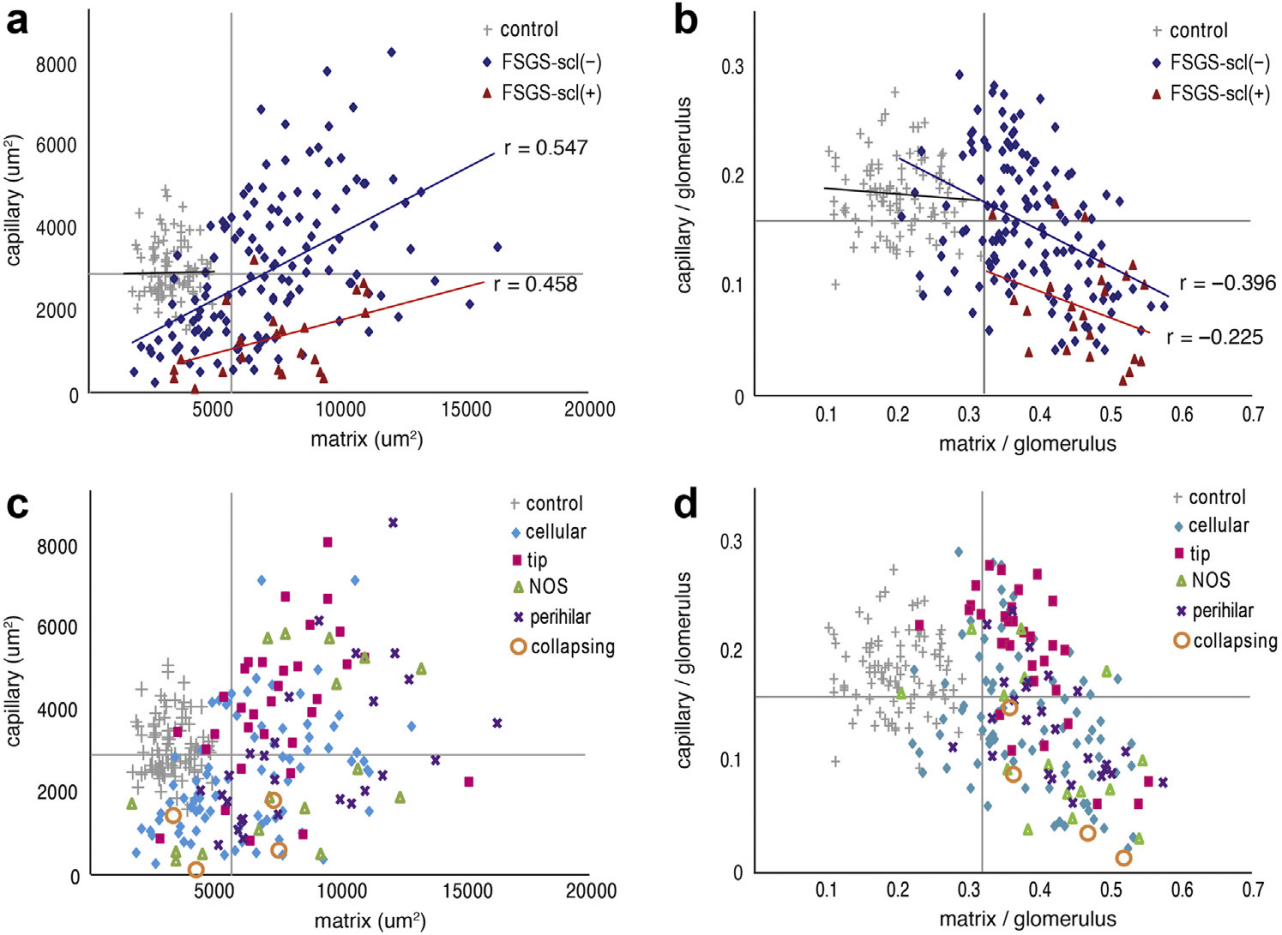
Association between glomerular capillaries and ECM area. Bivariate plots of glomerular capillary area and ECM area in 3 groups (FSGS-scl[+], FSGS-scl[−], and control glomeruli) are shown in (a). For bivariate plots of glomerular capillary/glomerular area ratio and ECM/glomerular area ratio made to avoid the effect of glomerular size, comparisons among the 3 groups (FSGS-scl[+], FSGS-scl[−], and control glomeruli) are shown in (b). Bivariate plots of the glomerular capillary area and ECM area in each FSGS variant are shown in (c). Bivariate plots corrected for glomerular size in each FSGS variant are shown in (d). All graphs are divided into 4 areas by drawing a horizontal or vertical line with the average value of all measured glomeruli. ECM, extracellular matrix; FSGS, focal segmental glomerular sclerosis; FSGS-scl(−), glomeruli without sclerotic lesions in FSGS; FSGS-scl(+), glomeruli with sclerotic lesions in FSGS; n.s., no significance; NOS, not otherwise specified; *r*, correlation coefficient, Spearman's rank correlation coefficient.

To identify whether there was a correlation between the area of both capillaries and ECM accumulation, we examined the results of computer-assisted image analysis using a bivariate plot (Figure 5a–d). All graphs were divided into 4 areas by drawing a horizontal or vertical line with the average value of all measured glomeruli. In the glomeruli of FSGS-scl(+), there were more distributions of smaller capillary areas and larger ECM areas than in the control group (Figure 5a). In contrast, many of the FSGS-scl(−) glomeruli showed a decrease in capillary area as in FSGS-scl(+). However, some glomeruli also showed increases in both ECM and capillary areas due to glomerular hypertrophy. There were significant positive correlation (FSGS-scl[−], *P* < 0.01, *r* = 0.547; FSGS-scl[+], *P* = 0.03, *r* = 0.458) between the area of the ECM and that of the capillaries in the glomeruli of FSGS. Furthermore, in comparison with the control group, significant positive correlation was observed in both FSGS-scl(−) and FSGS-scl(+) (control vs. FSGS-scl[−], *P* < 0.01, control vs. FSGS-scl[+], *P* = 0.02).

To avoid the effect of glomerular size, we further created a bivariate plot of capillary/glomerular area ratio and ECM/glomerular area ratio (Figure 5b). Even in correction for the glomerular area, there was a significant negative correlation between ECM area and capillary area in FSGS-scl(−) glomeruli (*P* < 0.01, *r* = −0.396). In the FSGS-scl(+) group, similar trend was shown (*P* = 0.312, *r* = −0.225), indicating that as the capillary area decreased, the sclerotic area (ECM) increased. Most of the FSGS-scl(+) glomeruli and many of the FSGS-scl(−) glomeruli fell into the lower right plot area, where the capillary area was smaller and the ECM area was larger than the average value. In addition, some FSGS-scl(−) glomeruli also fell into the upper right plot area where the ECM area increased. However, the capillary area was maintained compared with the control glomeruli. Of note, some FSGS-scl(−) glomeruli belonged to the lower left plot area and showed narrowed capillaries, even though the ECM was not increased. In other words, narrowing of the glomerular capillary might begin before ECM accumulation.

We then divided the plot by variant and checked data trends for each variant (Figure 5c). The capillary area of all glomeruli of the collapsing variant was smaller than that of the control group. In the upper right plot area, where both the capillary and ECM areas were larger than the average value, the glomeruli of the tip variant (65.6% of the glomeruli of the tip variant) were the most abundantly distributed, followed by the NOS (40.0%) and PH (34.6%) variants, with PH glomeruli being particularly conspicuous far from the intersection of the mean lines in this area.

Comparing each variant after adjusting for glomerular size (Figure 5d), the variation in the plots became less pronounced, all glomeruli of the collapsing variants and many glomeruli of the PH (63.0%), NOS (60.0%), and cellular (52.4%) variants belonged to the lower right plot region with small capillaries and large ECM areas, whereas the tip variant had the most glomeruli (65.6%) in the upper right region with large ECM areas and preserved capillaries.

**Figure 6:**
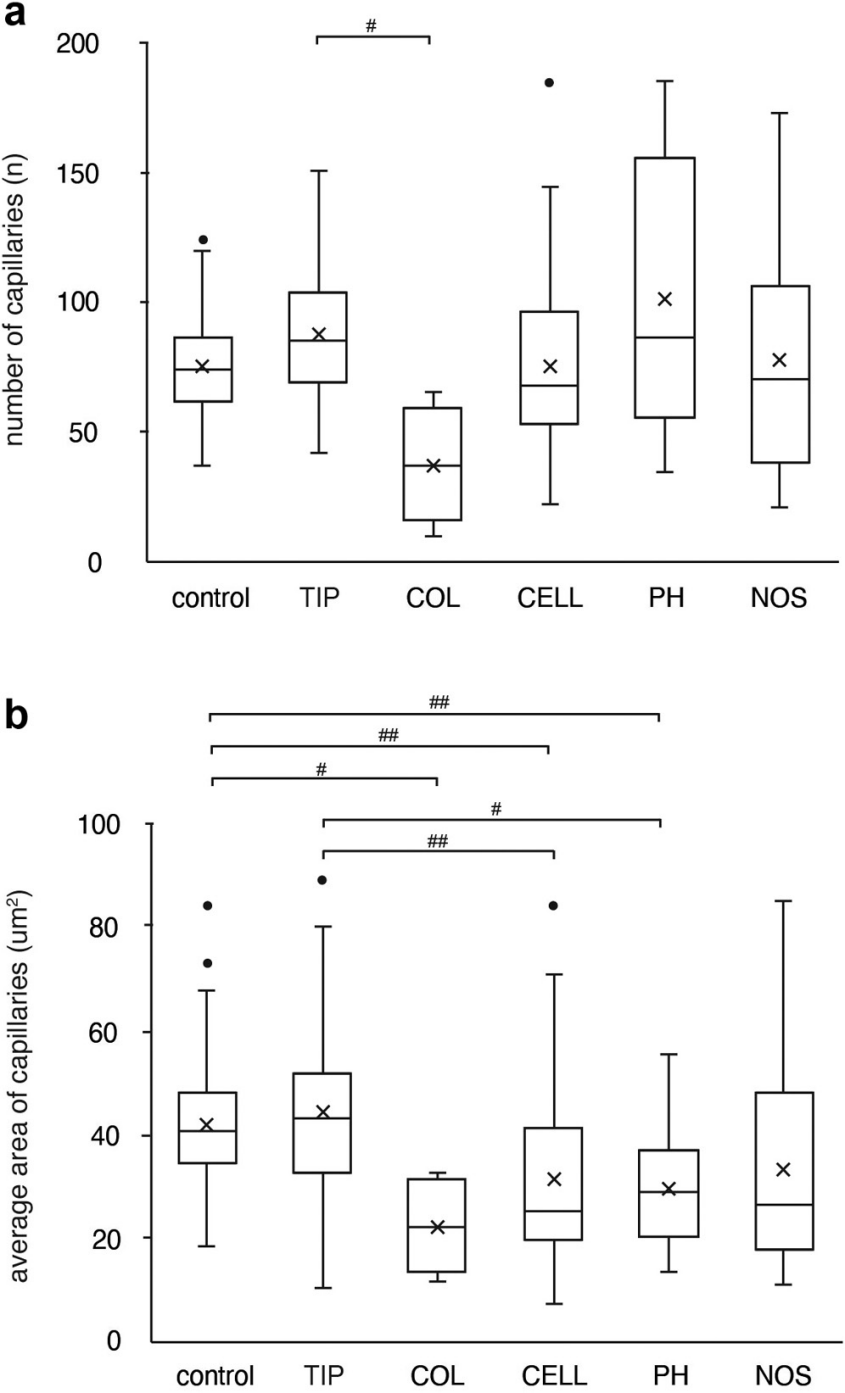
The quantitative analysis of glomerular capillary network in each variant of FSGS. The number of glomerular capillaries (a) and the average area of capillaries (b) in each variant and the control group. #*P* < 0.05, ##*P* < 0.01 Kruskal-Wallis test and for each group using Steel–Dwass test. The box corresponds to the first quartile, median (horizontal bar in the box), and third quartile, and the whiskers extend from minimum to maximum values. CELL, cellular; COL, collapsing; NOS, not otherwise specified; PH, perihilar; TIP, tip.

Subsequently, the average number and the average area of the glomerular capillaries were compared among all variants and the control group; Kruskal-Wallis test showed significant differences in both parameters (*P* < 0.01, respectively) (Figure 6a and b). The number of glomerular capillaries in the collapsing variant significantly decreased more than that in the tip variant by multiple comparison test (Figure 6a). Regarding the average area of the glomerular capillaries, there were also significant differences between the 2 groups (tip vs. cellular, *P* < 0.01; tip vs. PH, *P* = 0.02; control vs. cellular, *P* < 0.01; control vs. PH, *P* < 0.01; control vs. collapsing, *P* = 0.04). In comparison with each variant, capillary lumina narrowing and ECM accumulation were prominent in the collapsing variant, whereas capillary lumina narrowing was the least altered in the tip variant.

## Discussion

FSGS is a histopathologic term for a disease in which sclerotic lesions are found in part (segmental) of some (focal) glomeruli. It is characterized by podocyte injury, which can be caused by a variety of factors, including unknown circulating factors, genetic mutations, glomerular hyperfiltration, drugs, and infections.^1^ However, it is questionable why glomerulosclerosis is segmental even though all podocytes are equally exposed to abnormalities. Several studies evaluating FSGS specimens in serial sections or three-dimensional morphologic analysis have shown that changing sections often results in appearance of sclerotic lesions of the glomeruli that were normal in previous sections, suggesting that sclerotic lesions are actually present diffusely.^23^, ^24^, ^25^ Furthermore, in primary FSGS, there is widespread foot process effacement not only in the sclerotic lesions but also in the glomeruli without sclerotic lesions and in nonsclerotic areas of the glomeruli with segmental sclerosis,^26^ in which derangements in the glomerular epithelial cell phenotype preceded the FSGS lesions.^13^ In addition, in early stage of FSGS, foot process effacement without morphologically segmental sclerotic lesions, there have been reports that the proteome profile was different from normal in experimental models of FSGS.^27^ Moreover, the gene expression profile in human biopsy samples was different between minimal-change disease and FSGS.^28^ Thus, even in the glomeruli that have not yet developed sclerotic lesions on FSGS, podocyte injury and predisposition to sclerosis were present.

In contrast, there have been various reports focusing on EC damage in FSGS. These reports indicated that EC damage markers (circulating ECs, soluble thrombomodulin, and von Willebrand factor) were elevated in patients with FSGS.^29^ These reports also revealed that local podocyte loss was accompanied by thrombotic microangiopathy in a mouse model of podocyte-specific injury.^30^ In addition, reports on *in vivo* imaging of the FSGS puromycin aminonucleoside model showed that podocyte detachment resulted in local thrombi formation in the capillary loop directly underneath the shedding podocyte.^31^ Matsusaka *et al.*^32^ generated transgenic NEP25 mice, a model of acquired glomerular sclerosis by podocyte-specific injury. They reported that secondary damage to glomerular ECs and mesangial cells was observed due to severe podocyte injury, and swelling of ECs, mesangiolysis, and fibrin deposition were observed in areas of podocyte injury, which developed into FSGS.^32^ Menon *et al.*^33^ evaluated transcripts defining glomerular ECs that were assessed in biopsies from patients with various glomerular diseases. They reported that endothelial inflammatory status was higher in untreated patients with FSGS. In addition, they showed that the FSGS group with higher alpha-2 macroglobulin gene expression, a major downstream mediator of the EC phenotype, had a poorer prognosis.^33^ Furthermore, Sun *et al.*^34^ demonstrated that endothelial dysfunction and damage preceded podocyte injury using adriamycin-induced nephropathy model and they speculated that ECs had protective effect on podocytes.

In this study, we visualized and examined the alteration of glomerular capillary networks in FSGS. On EM, representative images of the glomeruli from patients with FSGS were characterized by loss of fenestrae, widening of the subendothelial space, swelling of the cytoplasm of the endothelium, and capillary lumen narrowing. The computer-assisted morphometric analysis showed that capillary lumen area was smaller and ECM area was larger in the FSGS glomeruli than in the control glomeruli, regardless of presence or absence of sclerosis. In addition, some FSGS glomeruli presented narrowed capillaries, even though ECM accumulation was not detected. Our results indicated the initiation of diffuse and global glomerular EC injury in FSGS glomeruli. Considering that EC injury progressed in conjunction with podocyte injury due to podocyte-EC crosstalk, it is suggested that ECs were injured in all FSGS glomeruli.

Furthermore, we assessed the morphologic alterations of the glomerular capillary network in each variant. Bivariate data analysis also showed that the glomerular tuft area was increased in some FSGS-scl(−) glomeruli compared with the control group. This occurred particularly in the PH variant, where glomeruli with large area far outside the intersection of the mean lines were observed. In secondary FSGS, PH variants are often seen in which glomerular hyperfiltration due to nephron loss and scarring leads to glomerular hypertrophy and podocyte stretching.^35^ However, glomerulomegaly has been reported to occur in 10% to 30% of patients with even primary FSGS.^26^^,^^36^ We suggested that some glomeruli, especially FSGS-scl(−) glomeruli, had glomerular hypertrophy. Therefore, image analysis was corrected for capillary/ECM area per glomerular tuft area. This correction allowed us to clearly identify the characteristics in each variant.

Quantitative analysis also showed that the glomeruli of the tip variants tended to maintain their capillary number and area, and EC injury was relatively mild in the tip variant consistent with EM findings. In contrast, in the collapsing variant, severe EC injury was observed with extremely narrowed and collapsed glomerular capillaries, and image analysis showed that the ECM was increased and the capillary was narrowed in all glomeruli.

Many reports indicated that remission rate and renal prognosis were the worst in the collapsing variant and the best in the tip variant.^3^^,^^5^^,^^6^^,^^9^ However, few studies have examined prognosis of FSGS, focusing on EC injury for each variant. Taneda *et al.*^19^ measured EC injury as subendothelial widening using electron micrographs of renal biopsies from patients with FSGS and reported its association with poor remission rate and decreased eGFR. These results suggest that severity of EC injury may reflect differences in prognosis of each variant. In this study, eGFR at the time of biopsy was also the lowest for the collapsing variant and the highest for tip among all the variants. However, the number of cases of each variant was very small and we could not assess their prognosis. Further research is needed. However, our result was consistent with the hypothesis postulating that there was an association between severity of EC damage and disease activity.

Regarding distributions of FSGS variants in this study, cellular was the most common and collapsing was the least common. Clinical studies on each variant of FSGS reported that NOS was the most common variant, and collapsing and cellular variants were the least common.^3^, ^4^, ^5^^,^^9^, ^10^, ^11^ Considering the possibility that all subtypes may become NOS variants as they progress to end-stage renal disease^1^^,^^37^ and cellular variants may be in the early stage of FSGS,^1^^,^^35^ the frequency of variants may vary depending on the timing of renal biopsy. The time to biopsy in the cellular variant in our study tended to be the second shortest after the tip variant, which tends to present with acute-onset NS. Furthermore, Stokes *et al.*^6^ pointed out that cellular variants may include unsampled collapsing and tip variants and discussed the need for additional tissue sections in all biopsies with features that appear to be cellular variants. In this study, the possibility of undersampling cannot be denied, and the relatively small number of cases at a single institution may have biased the variant.

What makes the difference in prognosis of FSGS and the boundary between repair and sclerosis is not well understood. However, various studies have shown that EC injury contributes to the process of sclerosis and may be a potential therapeutic target in the future. In the present study, we found presence of EC injury and diverse alteration of glomerular capillary networks in human renal biopsy samples from patients with FSGS. However, this was a retrospective study with a small number of patients at a single institution, and the analysis of each variant was limited by the small number of cases. Further studies are required to clarify the process of EC damage and sclerosis in patients with FSGS.

## Disclosure

All the authors declared no competing interests.

## Author Contributions

MM and AM conceptualized the study. MM, AM, and AS analyzed and interpreted the data. MM, FY, YA, and TK acquired the data. AM and AS performed the methodology. MM and AM drafted the manuscript. TK and AS supervised. All authors read and approved the manuscript.
